# Spiritual bypassing among Indian women: toward a culturally situated assessment framework

**DOI:** 10.3389/fpsyg.2026.1929993

**Published:** 2026-08-26

**Authors:** Maitri Patel, Navin Kumar

**Affiliations:** School of Social Sciences and Languages, Vellore Institute of Technology, Vellore, Tamil Nadu, India

**Keywords:** emotion regulation, feminist psychology, Indian women, religious coping, spiritual bypassing, transpersonal psychology

## Abstract

Spiritual bypassing — using spirituality to avoid unresolved psychological difficulty — has been studied almost entirely in Western samples. This conceptual paper asks whether the construct travels to Indian contexts and proposes a culturally situated alternative. The method is immanent critique: existing formulations are examined against their own stated aims to identify the assumptions they must make; those assumptions are tested against evidence from Indian cultural psychology, gender socialization research, and the psychology of religion. Literature is identified through targeted searches across three domains and synthesized thematically. Three assumptions are problematic in this context: that spiritual practice is freely chosen, that the practitioner has an individuated self, and that bypassing is intrapsychic. Three structural mechanisms are proposed instead: mythological archetypal conditioning as collective bypass scripts; religious language (karma, sahana, nishkama karma) as a spiritualized affect-regulation mandate; and seva as spiritually sanctioned self-erasure. These inform a three-dimensional assessment framework: Agency Gradient, Functional Directionality, and Archetypal Identification versus Individuation, each with indicators and clinical questions, and distinguished from constructs such as self-silencing, experiential avoidance, and religious coping. Although the factor structure of the Spiritual Bypass Scale (SBS-13) has replicated in an Indian sample, the gap addressed here is theoretical rather than empirical: no culturally grounded account specifies what bypassing consists of in this context. The framework is restricted to Hindu women in urban, middle-class settings shaped by the Vaishnava pativrata ideal, treats the mechanisms as patriarchal readings of religious traditions rather than as properties of those traditions, and states its claims as testable propositions.

## 1 Introduction

The question of when spiritual engagement facilitates psychological growth and when it obstructs it has long occupied transpersonal psychology. As a result, the concept of spiritual bypassing, originally introduced by Welwood (1984), has led to an extensive literature on the subject during the past four decades. However, what this body of work appears not to have accounted for thus far is that the construct itself may rely on theoretical presuppositions that are highly culturally specific. In other words, the assumptions behind current formulations about who the spiritual practitioner is, what kind of a self she possesses, and in what kinds of conditions she is able to engage herself in spirituality might be rooted in a specific culture and therefore might be insufficiently flexible to account for bypassing that occurs in different cultures.

According to Welwood (1984), spiritual bypassing occurs when spiritual beliefs, practices, and experiences are employed by individuals as a means of avoiding dealing with psychological issues, including unresolved issues stemming from childhood, interpersonal conflicts, or other developmental problems. Over the years, the concept of spiritual bypassing has attracted considerable scholarly attention in fields like counseling, transpersonal psychology, and clinical psychology. However, despite the ever-growing number of articles that have been written on the subject, the theory behind the construct has remained remarkably homogenous in terms of its cultural background. The assumptions inherent in current transpersonal approaches to spiritual bypassing imply that individuals freely choose spirituality and that they have an individuated enough sense of self to use spirituality as a tool for coping with psychological problems.

Such a perspective is likely to prove inadequate for examining contexts in which spiritual practices are not something people choose to pursue freely but are imposed by structural factors. Absent conceptual tools for examining structural spiritual bypassing, one that implies collective rather than individual choices, clinicians may be prone to misreading cultural affect suppression as religious coping, and researchers may find it correspondingly difficult to build a framework for their work. Thus, this paper aims at addressing this gap in theoretical understanding by doing the following. First, an immanent critique of existing theories of spiritual bypassing will be conducted to explore the hidden cultural assumptions behind them. Second, a discussion of three mechanisms that may promote spiritual bypassing among women in the specific contexts described below will follow. Finally, based on cultural psychology, feminist transpersonal psychology, and Pargament et al.’s (2013) theory of religious coping, the construct will be rethought in terms of a three-dimensional framework. It should be stated at the outset that the framework developed here is not offered as a general account of Indian women’s spirituality. The argument is built around, and is intended to apply to, urban, middle-class Hindu women whose lives have been shaped by the Vaishnava-influenced pativrata ideal; how far the same mechanisms extend to Muslim, Christian, Sikh, or Buddhist women, to Dalit and lower-caste communities, to rural populations, or to the diaspora is left open, and is addressed further in the Limitations section.

## 2 Approach and epistemological position

Because the analytic moves made in this paper are conceptual rather than empirical, it is worth being explicit about what kind of reasoning is being used, what counts as evidence for it, and what it can and cannot establish.

### 2.1 Immanent critique as method

The critique developed in the sections that follow is immanent rather than external: existing accounts of spiritual bypassing are assessed against the aim they set for themselves, namely, to distinguish spiritual engagement that supports psychological development from engagement that substitutes for it. The question is what a theory must assume for that distinction to do the work asked of it. Three assumptions emerge: that the practitioner elects her practice, that she possesses a self-sufficiently individuated self to be the one doing the avoiding, and that the avoidance is hers rather than her community’s. Each is then examined against what is documented about the circumstances of the women this paper addresses. Where an assumption does not hold, the consequence is not that the account is false, but that its range is narrower than it claims. Welwood’s clinical observations of Western practitioners are not in dispute here; what is in dispute is whether the diagnostic criteria built on them identify the same phenomenon when the practitioner’s circumstances differ in the ways described below.

### 2.2 Literature identification and synthesis

Literature was assembled through targeted rather than systematic searching, as the aim was theoretical synthesis across several bodies of work rather than exhaustive coverage of a single one. Searches were conducted in Scopus, Web of Science, Google Scholar, and area-studies indexes covering South Asian social science, using term clusters drawn from five domains: spiritual bypassing and its measurement; transpersonal theory and its cross-cultural critiques; emotion socialization and regulation in Indian samples; feminist and gender-role scholarship on Indian women; and religious coping in Hindu and South Asian contexts. Reference lists of retrieved sources were followed backward, and citation tracking was used forward until additional searching no longer surfaced materially new theoretical positions. Sources were retained if they contributed a theoretical position that the framework had to accommodate or contest, offered empirical evidence on the Indian populations under discussion, or documented conceptual developments in transpersonal and feminist psychology relevant to the argument. Given that a substantial part of the argument concerns cultural specificity, work by Indian scholars and studies drawing on Indian samples were deliberately prioritized over work generalizing to India from elsewhere. Synthesis was thematic: sources were grouped according to the assumption they bore on, and the three mechanisms proposed later in the paper emerged from recurring patterns across the cultural psychology, emotion socialization, and gender-role literatures rather than being imposed in advance.

### 2.3 Principles guiding framework construction

Four commitments shaped the construction of the framework. Dimensions were retained only where the existing literature provided a theoretical warrant for them and where the distinction they drew could not be captured by an existing construct—the test applied in the section on conceptual distinctiveness below. Each dimension was specified as a continuum rather than a category, on the grounds that a threshold model would require empirical calibration that this paper cannot provide. Each was required to be assessable through clinical inquiry rather than inference alone, which is why indicators are paired with assessment questions in Table 1. And each was required to be capable of returning a null or benign result, so that the framework can identify spiritual engagement as adaptive rather than only detect pathology. What follows is accordingly a set of theoretically derived propositions rather than findings. The framework has not been tested, and the claims about how the mechanisms relate to psychological outcomes are hypotheses that empirical work could disconfirm.

**Table 1:** Three-dimensional framework for assessing spiritual bypassing among urban, middle-class Hindu women.

Dimension	Theoretical basis	Operational indicators	Clinical assessment questions
1. Agency gradient (Coerced to Freely Chosen)	Markus and Kitayama (1991): interdependent self-construal Ferrer (2002): participatory spiritual engagement	*Sanction for non-participation.* What the woman reports would follow if she declined: nothing; verbal disapproval; withdrawal of family support; escalation to marital or economic consequence. *Entry route.* Whether the practice was taken up before adolescence without recall of a decision, adopted at a family transition (marriage, childbirth, bereavement) under expectation, or taken up independently in adulthood. *Revisability.* Whether the woman reports having ever altered, paused, or declined an element of the practice, and what followed when she did. *Articulated personal meaning.* Whether she can state what the practice does for her in terms independent of obligation, or accounts for it only by reference to duty or others’ expectations.	Did you choose this practice, or was it expected of you? What would happen — at home, or with people who matter to you — if you stopped or questioned it? Has there been a time you did it differently, or not at all? What followed? Setting aside what is expected, what does the practice do for you?
2. Functional directionality (Avoidant to Integrative)	Ferrer (2002): authentic development criteria Gross (1998): suppression vs. reappraisal Pargament (1997, 2011): positive vs. negative religious coping	*Affective range.* Whether anger, grief, and protest are reported as available and expressible, reported as felt but not expressible, or reported as absent — with attention to whether absence is described as attainment. *Naming alongside accepting.* Whether the woman can describe a situation as unjust while also accepting it, or whether acceptance appears to substitute for the appraisal. *Spiritual framing of self-neglect.* Whether karma, seva, or nishkama karma are invoked specifically at points where a need, grievance, or entitlement of hers would otherwise be stated. *Problem engagement.* Whether spiritual practice has accompanied any attempt to change a difficult circumstance, or has consistently accompanied accommodation to it. *Ferrer criteria.* Presence or absence of expanded self-knowledge, deepened relational capacity, and increased moral sensitivity over the course of the practice.	When you are hurt or angry, what do your beliefs tell you to do with that feeling? Is there anything in your situation you would call unfair? What do you do with that thought? Does your practice help you know what you need, or mainly help you set it aside? Since you began practising, what has changed in how you understand yourself and others?
3. Archetypal identification vs. individuation (Total Script to Reflective Agency)	Young-Eisendrath (1997): feminist individuation Rowland (2002): Jungian feminist revision Guzder and Krishna (1991): Sita-Shakti paradigm Daniels (2005): multi-vector transpersonal development	*Scope of identification.* Whether identification with Sita, the pativrata ideal, or a comparable figure is described as one valued aspect among several, or as what she fundamentally is. *Reflective distance.* Whether the woman can describe the ideal as something she was taught — naming a source, a time, or an alternative — or presents it as simply how things are. *Self-advocacy alongside care.* Whether she can name an instance of asserting her own need without framing it as failure, selfishness, or spiritual lapse. *Desire access.* Whether preferences and wants are reportable when asked directly, or whether the question returns blankness — distinguishing renunciation of desire from desire never having formed (Dhawan, 2005).	Which women, in stories or in your family, do you most identify with? What draws you to them? Where did you first learn what a good woman is? Did anyone around you see it differently? Can you think of a time you asked for something for yourself? How did that sit with you? If nothing were expected of you for a day, what would you want?

## 3 Theoretical background

### 3.1 Origins and development of the construct

According to Welwood (1984), the construct of spiritual bypassing originated from his observations of the way some contemplative practitioners used spirituality to sidestep dealing with their psychological material, using ideas about transcendence as an excuse not to cope with the challenges of human existence. In his follow-up study, Welwood (2000) advanced this idea and suggested that spiritual bypassing refers to a phenomenon of idealization of spiritual experience and preference for spirituality over psychological development and integration. Moreover, spiritual bypassing was defined as the use of certain characteristics, such as premature forgiveness, numbness towards unpleasant emotions, the adoption of attitude of universal positivity as a defense mechanism, and the separation of the spiritual desire from reality.

Another important dimension that must be considered when discussing the history of this construct involves its origin within the tradition of investigating the psychological impacts of spirituality or religiosity. Specifically, in what is considered a fundamental contribution to the psychology of religion, James (1902) stated that spirituality or religion should be judged not by the truthfulness of claims but by its impact on individual’s mental health and morals. In other words, spiritual bypassing can be regarded as a psychotherapeutic reformulation of the Jamesian criterion: rather than evaluating beliefs based on their truthfulness, the key question becomes the effect that spirituality has on people. The present paper accepts the functional criterion proposed by James. Nevertheless, it argues that both the Jamesian criterion and subsequent formulations of spiritual bypassing were developed on the assumption that conditions associated with a liberal-individualist worldview prevailed in the context in which the construct emerged. Although these assumptions may appear self-evident within that sociohistorical context, they must be taken into account if the concept is to be applied to different social realities.

Over the years, the number of empirical studies on spiritual bypassing has increased steadily; however, they continue to rely on the original definition proposed by Welwood (2000). The first empirical study of spiritual bypassing, based on a sample of clients in Western counselling settings, was conducted by Cashwell et al. (2010). To facilitate research on the construct, Fox et al. (2017) developed and validated the Spiritual Bypass Scale-13 (SBS-13), which remains the most widely used measure of spiritual bypassing. The scale has shown to be composed of two factors, namely Psychological Avoidance and Spiritualizing, and higher SBS-13 predicted the presence of depression and anxiety symptoms (Fox and Picciotto, 2019). Cross-cultural research has also become increasingly common. Motiño et al. (2021), for example, found considerable differences between cultural groups in the associations between spiritual bypassing and psychological outcomes. Adaptation work has extended to Brazil (Picciotto et al., 2018) and, importantly for the present argument, to India. Toussaint et al. (2021) administered the SBS-13 to 250 English-speaking Indian college students, most of whom were women and Hindu, and replicated the two-factor structure previously identified in the United States and Brazil, with alpha coefficients above 0.80.

That finding matters for how this paper’s contribution should be understood, and it narrows the claim being made. The gap addressed here is not that spiritual bypassing has never been measured in an Indian sample—it has, and the instrument performed well psychometrically. The gap is theoretical. Structural replication of a factor solution establishes that the items cohere in a new population; it does not establish that they capture what spiritual bypassing consists of in that population, still less that they capture the gendered and religiously sanctioned processes described in this paper. The SBS-13 asks about spiritualizing and psychological avoidance in general terms. It does not ask whether a woman’s anger has been reframed as karmic debt, whether her self-neglect is understood as *seva*, or whether the self that would be doing the avoiding was permitted to form. A measure can replicate cleanly and still remain blind to the phenomenon of interest. What is missing, then, is not psychometric groundwork but a culturally grounded account of the construct on which such groundwork could be built, which is what the present paper attempts.

### 3.2 Limitations of existing frameworks

#### 3.2.1 The voluntarism problem

Welwood’s (1984, 2000) conceptualization of spiritual bypassing, along with Masters’ (2010) subsequent clinical elaboration, largely frames spiritual practice as a voluntary coping strategy adopted by autonomous individuals in response to psychological distress. This assumption becomes less defensible in the contexts examined here, For the women considered in this paper, religious practices, spiritual obligations, and moral ideals are often part of their everyday life due to family socialization and community norms. They may also be reinforced by the possibility of social or moral disapproval. Therefore, spiritual engagement may reflect personal conviction while also being shaped by expectations linked to women’s social roles.

Furthermore, empirical evidence supports this argument. In a study, Raval and Martini (2009) found that Indian mothers were more accepting of anger expression in sons than in daughters. This finding suggests that gendered expectations about emotional expression may begin to develop early on childhood. Although spirituality was not the focus of their investigation, the findings nevertheless indicate that emotional regulation develops within broader systems of gender socialization. These same processes help shape the expectations and obligations through which women’s engagement with spirituality is understood and enacted.

One implication deserves particular attention because it shapes the limits of what any intervention can achieve. If engagement with spirituality is experienced as a normative expectation rather than an individual choice, addressing patterns of spiritual bypassing may require more than psychological insight alone. It may also require women to bear the interpersonal and moral costs of departing from behaviours and values that their communities regard as virtuous.

#### 3.2.2 The individualist subject problem

Welwood and Masters both locate the unit of analysis in the individuated self. The notion of personhood that is assumed in this perspective is, of course, not universally applicable. It is a culturally based concept within the traditions of Western psychology that cannot be easily applied to other sociocultural settings. Markus and Kitayama (1991) distinguished between independent self-construals, typically associated with Western societies, and interdependent self-construals, in which the self is fundamentally constituted through relationships, social roles, and obligations. Kakar (1982) similarly argued, from a psychoanalytic perspective, that Indian selfhood tends to be organized around relational embeddedness rather than autonomous individuation. Consequently, behaviors that Western frameworks might classify as manifestations of spiritual bypassing such as subordinating personal needs to relational responsibilities or accepting suffering as an expression of duty may instead represent normatively sanctioned and culturally consonant forms of experience, rendering them largely invisible to assessment instruments developed for individualist conceptions of the self.

#### 3.2.3 The absence of structural analysis

Neither Welwood nor Masters considers the possibility that spiritual bypassing may be collectively produced — sustained through shared meanings, normative expectation, and institutional arrangement rather than through individual defence. Ferrer (2002) challenged the perennialist and universalist presuppositions that underlie transpersonal psychology by disputing the idea of a single developmental path that remains constant regardless of cultural differences. Instead, he advanced a participatory approach that understands spiritual experiences as contextual, relational, and co-constructed. The present paper continues with the critique of the perennialist assumptions made by Ferrer (2002), but from a feminist and postcolonial perspective, which highlights that an understanding of spirituality necessarily requires attending to how it is shaped by both the material and symbolic contexts within which it exists.

Patriarchal values, caste structures, and the myths surrounding femininity are not merely the social contexts within which spirituality unfolds; they actively shape spiritual practice and influence how suffering itself is understood (Patel, 2012).

### 3.3 Agency within structure, and the bypassing/coping distinction

The risk of essentialism inherent in all structural analyses lies in the possibility that it will portray women as passive objects of the operation of social forces instead of active participants who negotiate, reinterpret, and resist their culturally conditioned environment. This is not what will be proposed here. The women described in this paper are actors who appropriate, question, and rework the meanings attached to culture and spirituality, in varied ways and within particular circumstances (Phadke et al., 2011; Nair, 2020). Thus, it will be assumed that the range of spiritual engagement in question is from structural constraint to appropriation and not from coercion to autonomy.

In addition to the issue of categorization mentioned above, there is another related problem identified by Picciotto and Fox (2018). Namely, spiritual bypassing should be differentiated from authentic spiritual coping. From the perspective of clinical psychology, the latter can be regarded as a valid use of spirituality in managing negative experiences. The belief in reincarnation and karma, for instance, may allow the individual to make sense of trauma and promote emotional regulation and growth (Anand, 2009). At the same time, it may also serve as a way of avoiding acknowledgment of one’s right to self-defense and processing difficult emotions.

Pargament’s (1997, 2011) framework of religious coping provides one of the most useful existing approaches for navigating this distinction. His differentiation between positive religious coping which involves benevolent religious reframing, a felt sense of spiritual connection, and collaborative engagement with the sacred and negative religious coping, characterized by spiritual discontent, punishing attributions, and a sense of spiritual abandonment, maps directly onto the functional question this paper is trying to answer: not what a woman believes, but what her beliefs are doing for her psychologically. His later work on sacred loss and desecration (Pargament and Mahoney, 2005) is particularly germane to contexts in which a woman’s own spiritual framework is used to interpret her suffering as spiritually productive. Two qualifications matter. The framework was developed within Abrahamic traditions, though adaptation work exists, Tarakeshwar et al. (2003) built and piloted a Hindu religious coping measure, discussed further below. More consequentially, the positive/negative distinction sorts by the valence of a person’s relationship to the sacred, which is not the axis at issue here — a point developed in the section on conceptual distinctiveness. What does transfer is the underlying functional question: does this spiritual engagement open psychological possibilities or foreclose them?

The Functional Directionality dimension developed below is this paper’s answer to that distinction. Having outlined where existing theory falls short and how spiritual bypassing can be distinguished from authentic religious coping in principle; the discussion now turns to the specific mechanisms through which structural spiritual bypassing is produced and sustained in the everyday lives of the women considered here.

## 4 Three proposed mechanisms of gendered spiritual bypassing in urban Vaishnava-influenced contexts

### 4.1 Locating the mechanisms: doctrine, interpretation, socialization, institution

Before the mechanisms are described, a distinction needs to be drawn that the spiritual bypassing literature has generally left implicit, and that matters a great deal here. Four things can be confused with one another: the doctrinal content of a religious tradition, the interpretations of that content that circulate in a given community, the family and community practices through which those interpretations are transmitted, and the institutional arrangements that give them force. The claim advanced in this paper is located at the second, third, and fourth of these — not the first.

Consider the doctrine of *nishkama karma*, or action performed without attachment to its fruits. Nothing in the Bhagavad Gita’s formulation of this doctrine is gendered, nor does it require that a woman endure mistreatment without complaint. What is described later in this paper is a particular interpretation of the doctrine, one in which detachment is selectively prescribed to women in domestic contexts and in response to grievances that a differently situated person would be entitled to express. The doctrine itself should not be understood as the mechanism. Instead, the mechanism lies in how the doctrine is selectively interpreted and applied, particularly when these interpretations are shaped through family socialization and reinforced by material dependence that makes departure costly. The same distinction applies to karma and seva. Both concepts allow for a range of interpretations, many of which extend beyond their use as bypass scripts. The present argument therefore concerns these narrower interpretations and applications, rather than karma, seva, or the doctrines themselves.

Hindu traditions containing resources for challenging these interpretations is central to the argument and should be established in earlier rather than introduced later as a qualification. The Bhakti movements offer some of the clearest examples. For instance, Mirabai’s devotion to Krishna functioned as a rejection of the obligations imposed on her as a wife and daughter-in-law. She expressed this refusal through religious devotion rather than in opposition to it (Sangari, 1990). Likewise, Akka Mahadevi rejected marriage and prevailing social conventions, using her *vachanas* to portray conventional femininity as an obstacle to, rather than a path toward, union with Shiva (Ramanujan, 1973; Chaitanya, 2017). In fourteenth-century Kashmir, Lal Ded’s poetry similarly challenged both Brahminical orthodoxy and the social expectations placed on women (Hoskote, 2011). Importantly, none of these women positioned themselves outside the traditions in which they lived. Rather, each is remembered and venerated within those traditions (Ramaswamy, 2007).

Two qualifications are needed. Much of what is popularly known about these women comes from hagiographies composed long after the poetry attributed to them, and Pechilis (2023) argues that interpreting female *bhakti* saints through their medieval biographies rather than their verse has distorted the scholarship. The claims made here therefore concern what the poetry does, rather than questions of biographical fact. Sangari (1990) further cautions against treating Mirabai as an unambiguous figure of resistance, arguing that her *bhakti* was simultaneously radicalizing and compensatory. That ambivalence is precisely the point. The same devotional vocabulary could serve either refusal or accommodation, depending on how it was taken up, which is the distinction the framework developed below attempts to make assessable. The mechanisms described in what follows should therefore be understood as one interpretive settlement among several that the tradition permits—the settlement dominant in the contexts under discussion—rather than as an account of what Hindu spirituality does to women.

### 4.2 Mythological archetypal conditioning as collective bypass scripts

Hindu mythological archetypes seem to operate as psychological templates for femininity, acquired through socialization within families and communities, formal education in religious contexts, and cultural narratives in general. As has been argued by Sutherland (1989), characters such as Sita, Savitri, and Draupadi seem to convey a set of implicit expectations regarding the endurance of suffering, subordination of personal desires to family duties, and feminine virtue as a state of forbearance and self-sacrifice. Guzder and Krishna (1991), drawing on clinical case material, suggested that the archetype of Sita-Shakti could be employed by women in these settings to frame their sense of selfhood. Within the mythological paradigm examined here, Sita represents an idealized model of femininity characterized by suffering, devotion, humility, and self-deprecation. Her portrayal may therefore shape culturally approved expectations of womanhood, including how women view themselves, make sense of hardship, and respond to adversity.

The mythic traditions of Hinduism should not, however, be understood as a single or uniform system. Indian women have access to a diverse range of devotional archetypes, shaped by differences in region, caste, and devotional tradition. Andal, for example, represents a form of devotion that is passionate, personal, and active. Her intense longing for the divine and determined desire for union with God differ sharply from the Sita- type paradigm. Draupadi also does not fit easily within such a framework. Her anger following the dice game marks an important moral turning point in the Mahabharata, while her repeated demands for justice challenge an interpretation of her as simply an embodiment of patience and forbearance. Singh (2023) further suggests that, although the Sita and Kali archetypes offer different forms of feminine identification, both remain shaped by broader ideas of nurturant feminine virtue. However, Kali challenges any straightforward association between femininity and quiet endurance. This contrast highlights the range of archetypal possibilities available within Hindu traditions. Furthermore, Dube’s (1988) analysis of gender construction in Hindu patrilineal India similarly highlights that these archetypal templates are unevenly encoded across caste contexts, with upper-caste Vaishnava norms especially privileging ideals of purity, self-sacrifice, and subordination that are not uniformly shared across Hindu communities. The mechanism described in this paper is therefore most applicable to contexts shaped by the Vaishnava *pativrata* ideal and should not be generalized beyond such contexts without empirical investigation.

The psychological consequence of mythological archetypal conditioning can be defined as collective bypass scripts. To clarify this concept, it must be contrasted with existing notions such as schema. In Bem’s (1981) analysis, a gender schema represents a psychological template that organizes perception and behavior according to culturally defined categories. However, the proposed concept focuses specifically on the spiritual nature of the process under discussion. The suffering that is prescribed by an archetypical model is spiritually rather than socially endorsed. Therefore, deviation from it is not seen as a social transgression but a spiritual failure.

Collective bypass scripts should also be distinguished from Bourdieu’s (1977) notion of habitus because the latter emphasizes the embodied reproduction of social structures, while bypass scripts refer explicitly to religious narratives that attribute spiritual significance to the suppression of negative affect. Consequently, the proposed construct seems more precise because it addresses the specific way in which culturally available spiritual stories preemptively assign spiritual virtues to painful experiences, thus making anger inappropriate.

Observations can serve as supporting evidence for the suggested conceptualization. De Sousa and De Sousa (2015) reports that the subjection of women’s identity to norms of self-sacrifice and devoted family service co-occurred clinically with somatization and conversion disorders, though the observational nature of this work does not establish that the former produces the latter, a pattern supported by theories of spiritual bypassing. In the cases reviewed by Guzder and Krishna (1991), one woman understood her multiple experiences of physical violence against her within the framework of prescribed suffering, which was considered a spiritual duty. Her condition that includes somatic complaints, suppressed affect, and a notable absence of anger could be clinically explained only with reference to Sita identification, which turned suffering into something spiritually and morally meaningful, thus creating barriers to its expression and resistance.

### 4.3 Instrumentalized religious language as an affective suppression mandate

The second pathway refers to the instrumentalization of various philosophical terms and ideas inherent to Hinduism. Karma (consequences of moral conduct across lives), sahana (the value of forbearance), and nishkama karma (desireless action) could serve as some illustrative examples of concepts within which negative affect may be regulated. Within their original philosophies, each idea embodies elaborate and highly refined ethical principles. However, within patriarchal societal arrangements, each idea can also be used as coercive discourses that would discourage women from expressing emotions. Women’s anger in reaction to violence perpetrated against her might be viewed as her karma and thus something that she needs to accept. Grief related to her social position may suggest that a woman has not been able to detach herself sufficiently from her circumstances.

Furthermore, Anand’s (2009) study of women experiencing major personal crisis highlights this ambivalence. Participants primarily drew on karma as a way of understanding their experiences, and this framework appeared to serve an adaptive function by providing meaning, coherence, and continuity during periods of distress. However, the same framework may also function as a mandate for emotional suppression, making it more difficult for women to acknowledge and express what they are feeling. Therefore, this paper introduces the term *spiritualized affect regulation mandate* to describe this process. Gross (1998) conceptualized expressive suppression as a strategy used by individuals to regulate their emotions. Collectively, the same process can be understood through suppression norms, which refer to culturally shared expectations about which emotions should be expressed in social contexts. The proposed term builds on this idea by adding a spiritual dimension. Where a suppression norm renders emotional expression socially inappropriate, a spiritualized affect regulation mandate renders it spiritually illegitimate, framing it as evidence of insufficient virtue or inadequate spiritual practice. Two consequences follow. First, non-compliance carries theological as well as social consequences, making the norm more difficult to resist. Second, the psychological costs of suppression become more difficult to recognize because what is suppressed is experienced not as a restriction, but as a spiritual accomplishment. Suppression thereby migrates out of the vocabulary of emotion regulation and into the vocabulary of spiritual accomplishment.

The broader body of research in emotional regulation provides useful insights, although there is room for improvement in this area, as well. Gross (1998) demonstrated that expressive suppression compared to cognitive reappraisal was associated with worse psychological functioning and greater psychological distress. Later, Kathuria et al. (2023) found that Indian girls compared to boys were socialized to suppress negative emotions to a much larger extent. Although both pieces of evidence indirectly point towards the possibility of religion-related emotional regulation, the former cannot confirm such associations due to lack of cultural and demographic representation, while the latter does not address the religious dimension directly.

Several strands of recent empirical work bear on the present argument, although none tests it directly. On the question of gendered suppression, Iqbal et al. (2025) surveyed 304 Indian women aged 25 to 35 and found that family conformity orientation—the extent to which families emphasize deference to authority and agreement over open disagreement—predicted self-silencing, which in turn predicted depressive symptoms. Their model is structural in the sense relevant to the present argument: it traces the pathway from the family communication environment to an intrapsychic pattern and, ultimately, to a clinical outcome, rather than treating the problem as residing within the woman alone. Similarly, Emran et al. (2023) found that self-silencing mediated the relationship between attachment orientation and depression in Indian samples, with gender moderating this relationship. However, neither study considers whether spiritual framing changes this association and it remains unclear whether a woman who understands her silence as a religious accomplishment shows the same relationship with depression as a woman who experiences it as a form of relational accommodation. This is the central proposition advanced by the present framework, and it can be examined using existing instruments.

Research on how Indian women understand and make sense of their distress points in a similar direction. In interviews with women in Goa, Pereira et al. (2007) found that depression was understood primarily in terms of social and relational circumstances, including marital difficulties, family conflict, and economic strain, rather than as an illness. As a result, women expressed their distress mainly through social rather than psychiatric terms. If the language used to express distress is shaped by culture, it is therefore important to consider what a spiritual vocabulary allows women to express and what it may leave unspoken.

The broader disciplinary context for this argument is feminist psychology in India, whose development provides an important context for the present discussion. In her review of two decades of psychological research on gender, Vindhya (2007) identified three areas that had received sustained scholarly attention: the work-family interface, mental health, and violence against women. This observation is particularly relevant to the present argument. Research on the work-family interface, she notes, has been pursued largely within mainstream psychology and carries a sample bias towards urban middle-class women, while the more critical work on mental health and domestic violence has come from cross-disciplinary engagement between academics and advocacy groups rather than from the discipline proper. Spirituality appears in none of the three areas as a topic in its own right, which is part of what this paper is responding to.

Two features of that history bear directly in the present argument. The first is a caution the paper must apply to itself: if mainstream Indian gender research has over-sampled urban middle-class women, then a framework built substantially on that literature inherits the bias, and the scope restriction stated earlier is not merely a limitation of the framework’s reach but a reflection of where the evidence base actually is. The second concerns marginality. When gender first appeared in the Indian Council of Social Science Research’s decadal surveys of psychology, the chapter’s title reflected the subject’s status within the discipline—*On the Periphery* (Bharat, 2001). Davar’s (1999) argument that Indian women’s mental health required an explicitly feminist agenda, rather than incorporation into existing psychological categories, was made in response to that same marginality. Kapadia’s (1999) conceptualization of gendered socialization and the self similarly articulates a position central to the present paper: that a woman’s sense of self is constituted through the socializing arrangements within which she is situated, rather than merely influenced by them.

The mechanisms proposed in this paper occupy an intersection that these literatures have largely left unexplored. Feminist psychology in India has documented the ways patriarchal arrangements shape women’s psychological lives, while Indian psychology has developed sophisticated accounts of spiritual and contemplative experience. The two, however, have rarely been brought into conversation with one another. Where spirituality does appear in feminist scholarship, it is more often treated as a site of resistance, as in work on the Bhakti traditions, than as a possible vehicle of constraint. Both possibilities exist, and the framework developed here seeks to specify the conditions under which one or the other becomes operative.

The literature on religious coping is more developed than earlier sections of this paper suggested. Tarakeshwar et al. (2003) developed and piloted a measure of religious coping among Hindus, finding it to be a salient construct associated with better mental health and identifying Hindu-specific forms of religious coping—including karma-based and detachment-oriented strategies—not captured by the general RCOPE. Their finding that Hindu respondents reported relatively infrequent use of negative religious coping is particularly noteworthy because, from the perspective developed here, it admits of more than one interpretation. It may reflect genuinely adaptive religious engagement, or it may indicate that the construct of negative religious coping—built around spiritual discontent and punishing attributions—does not capture the form of difficulty at issue in the present paper. Distinguishing between these possibilities requires the functional, rather than valence-based, criterion proposed by the present framework.

However, even the first study should not be regarded as conclusive. Matsumoto et al. (2008) found that, in some collectivist societies in East Asia, expressive suppression was associated with lower levels of negative emotion and greater psychological well-being. These findings suggest that caution is warranted before treating suppression as an inherently maladaptive strategy. At the same time, the cultural and psychological differences between East and South Asia, particularly the differing roles of religion, make these forms of collectivism difficult to compare in relation to the present argument.

What the East Asian literature does demonstrate is that the relationship between expressive suppression and psychological morbidity is not culturally invariant. Further empirical exploration of the problem in Indian population is necessary to develop valid conclusions about the impact that suppression may have under specific socio-cultural conditions. The Functional Directionality dimension of the suggested assessment framework may help to achieve this goal.

When spiritual vocabularies start becoming the dominant means of interpreting and expressing one’s emotions, psychological costs of suppression may get incorporated into the stories of spiritual accomplishment. To illustrate this dynamic, one can refer to some findings from Anand’s (2009) work. For example, statements like, *“This was written for me,”* do not only indicate spiritual meaning-making in the face of adversity but also imply affective closure and premature cessation of mourning before emotions have been adequately acknowledged and processed. The issue, thus, is not the use of spiritual concepts to make sense of one’s suffering but its potential to prevent such suffering.

### 4.4 Seva as interpreted obligation: spiritually sanctioned dissolution of self

Within the interpretive framework described at the beginning of this section, seva is positioned as the highest form of spiritual aspiration considered available to women. That positioning has important implications for what this paper conceptualizes as spiritually sanctioned self-erasure—the attenuation of individual needs, aspirations, and psychological boundaries in the pursuit of spiritual virtue. Dhawan (2005) argued that prescriptive relational identities as daughter, wife, mother, and daughter-in-law may represent a substantial impediment to individuated identity development among the women in question. When this subordination is further valorized as the woman’s highest spiritual aspiration, it may acquire a sacral legitimacy that makes questioning it both socially transgressive and spiritually suspect.

It is theoretically important to distinguish spiritually sanctioned self-erasure from transpersonal ego dissolution, though doing so requires navigating a genuine tension in the framework’s theoretical resources. Wilber’s (2000) pre/trans distinction — which differentiates pre-egoic and trans-egoic states in terms of the presence and absence, respectively, of a formed self — offers a clinically useful descriptive tool. Ferrer (2002), whose participatory critique this paper otherwise follows, has challenged Wilber’s developmental hierarchy as imposing a normative sequence that does not apply universally. The present paper draws on the former but not the latter aspect of Wilber’s model: the distinction between a self that is consciously transcended and one that was never formed in the first place does not require commitment to a universal developmental sequence. It requires only observation that transcendence and foreclosure are functionally distinct. Washburn (1994) described ego dissolution as a post-individuation phenomenon requiring prior establishment of a stable personal self; spiritually sanctioned self-erasure, by contrast, appears to prevent such selfhood from forming in the first instance. Young-Eisendrath (1997) found that individuation from gender-role prescriptions was a prerequisite for transpersonal ego transcendence.

A theoretically more flexible grounding for this distinction is available in Daniels’ (2005) multi-vector model of transpersonal development. Where Wilber posits a single hierarchical developmental sequence through which all individuals must pass, Daniels proposes that transpersonal experience can be accessed through multiple, non-hierarchical vectors — including vectors oriented toward self-transcendence, toward depth of interiority, and toward relational dissolution. This is directly relevant to the present argument because it allows the distinction between spiritually sanctioned self-erasure and genuine transpersonal ego dissolution to be maintained without invoking a normative developmental sequence. On Daniels’ account the relevant question concerns the quality of a given selflessness rather than its rank on a developmental scale: was the self set aside once formed, or was its formation foreclosed? Framed this way the distinction is functional and phenomenological rather than hierarchical and developmental, which sits better with the cross-cultural commitments of this paper.

This idea is illustrated vividly by Dhawan’s (2005) observations in her work with clients suffering from depression and identity diffusion. In describing their selflessness, the patients spoke of a state in which they felt like blanks and devoid of desires since no personal preferences and wants had been ever developed by them as a possibility of subjective experience. This means that, far from being a transcendent spiritual virtue, the condition in question should be understood as an outcome of an inability to develop a self. In this context, a woman’s lack of desires is a disguise covering up a far more profound issue: the very absence of personal self-experience. As far as gender-related aspects of spiritual bypass are concerned, the above definition of the phenomenon can shed light on the situation when therapists fail to see the problem because of the influence exerted by cultural stereotypes on their perception. Since self-effacement is typically considered a female spiritual virtue, practitioners are prone to take a client’s selflessness as an indication of successful ego transcendence. According to Daniels’ approach, such an attitude is problematic from the point of view of developmental phenomenology. Each of the three mechanisms described above operates at the interpretive and socializing levels distinguished at the outset of this section rather than at the doctrinal one: what is transmitted is a reading, what sustains it is family and community practice, and what gives it force is the material dependence that makes departure costly.

**Figure 1:**
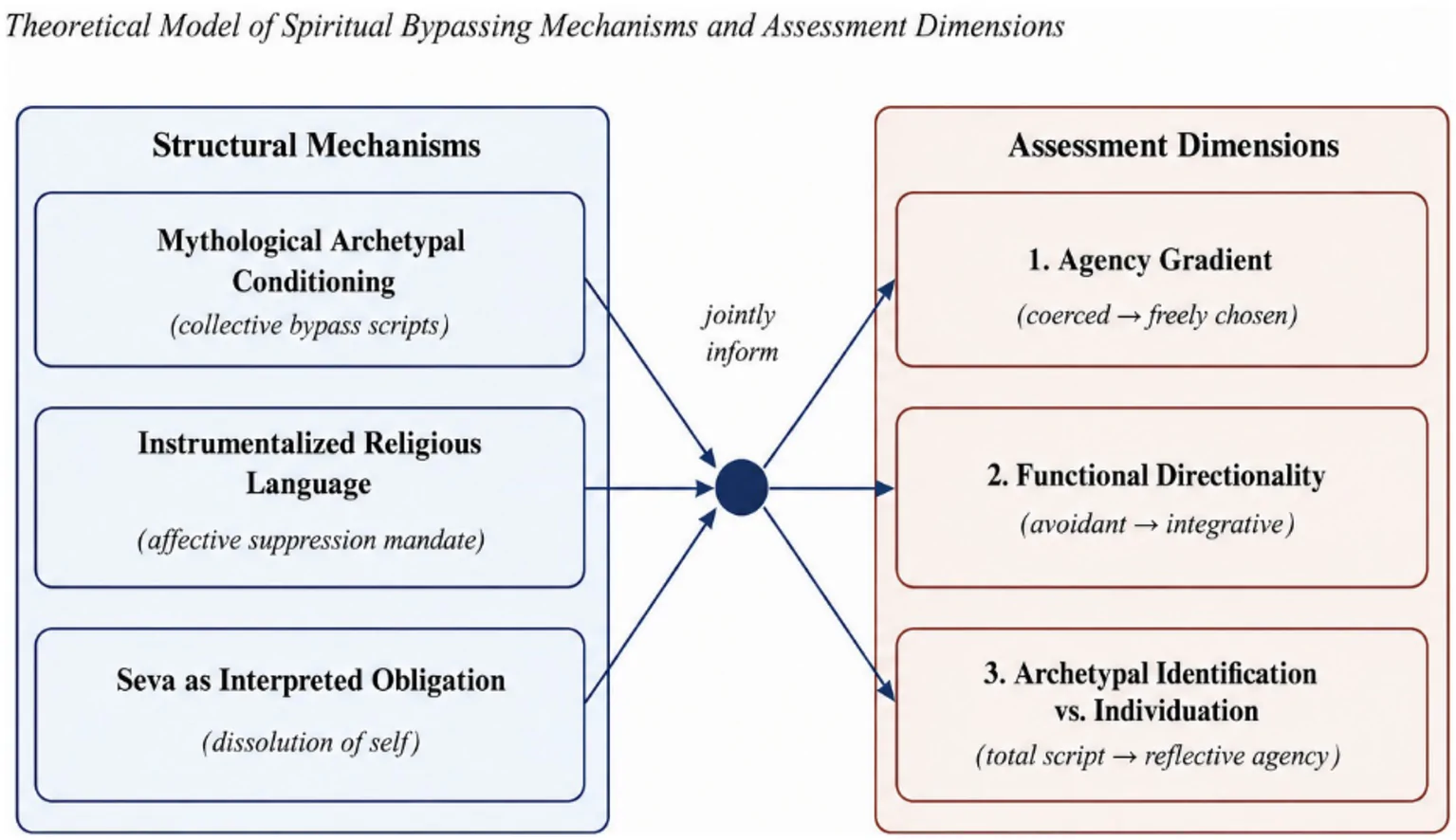
Conceptual relationship between the three structural mechanisms and the three-dimensional assessment framework. Each mechanism is theorized to inform a woman’s standing on all three dimensions jointly, rather than mapping one-to-one onto a single dimension.

Having described these three mechanisms separately, it is worth being explicit about how they relate to one another and to the assessment framework introduced next. They are not conceived as three independent causes operating in isolation, but as interrelated processes. Archetypal conditioning provides the cultural script, spiritualized affect regulation mandates regulate the emotional expression that might otherwise disrupt that script, and seva provides the behavioural means through which the script is enacted in everyday life. Figure 1 summarizes this relationship and shows how each mechanism feeds into the three assessment dimensions developed below.

## 5 Towards a culturally situated framework: the three-dimensional model

A three-dimensional, culturally situated framework is proposed to facilitate the understanding and assessment of spiritual bypassing among the women whose circumstances this paper addresses. Each dimension is grounded in the theoretical arguments developed throughout the paper, carries potential clinical relevance, and is operationalized through the indicators and illustrative assessment questions presented in Table 1. The framework is intended to serve as a heuristic tool to be used for further research, and not as an infallible classificatory tool. Therefore, it is best viewed as one consisting of continua that indicate varying degrees of spiritual behavior rather than as distinct categories.

### 5.1 Operationalizing the dimensions

The indicators in Table 1 refer to what a clinician or researcher would hear rather than to states inferred from outside: a consequence the woman anticipates, an occasion she can recall, a distinction she can or cannot draw. Writing them this way has a consequence worth stating, since it runs against how deficit-oriented assessment usually works. Each indicator is constructed so that its absence carries as much information as its presence. A woman who can name an occasion when she declined a practice, or who answers a direct question about what she wants, has supplied positive evidence of standing high on the relevant dimension — not merely failed to supply evidence of standing low on it.

Table 2 describes what each dimension looks like at its low pole, at an intermediate position, and at its high pole. The anchors are descriptive reference points and candidate content for item development; they are not a scoring scheme, and whether the poles are empirically separable as described remains to be established. Nothing in them is diagnostic on its own. A woman standing at the low pole of the Agency Gradient has not thereby been identified as bypassing: low agency accompanied by integrative directionality describes a woman whose practice was not chosen but is nonetheless doing her good, which is a different clinical situation and calls for a different response.

**Table 2:** Provisional anchors for the three dimensions.

Dimension	Constrained	Mixed	Unconstrained
Agency gradient	Coerced. Practice began before recall or at a family transition; declining is reported as carrying material or relational consequence; no instance of alteration; meaning stated only as duty.	Conventional. Practice adopted through socialization and sustained by expectation, but some personal meaning is articulable and minor variation has occurred without serious consequence.	Appropriated. Practice has been examined, altered, or suspended at some point; the woman can state what it does for her independent of obligation; declining is not reported as costly.
Functional directionality	Avoidant. Anger and grief reported as absent or spiritually inappropriate; injustice not nameable alongside acceptance; karma or seva invoked precisely where a need would be stated; no instance of the practice accompanying attempted change.	Mixed. Affect accessible in some domains and foreclosed in others; injustice nameable but rarely acted on; practice supplies meaning and, in places, forecloses response.	Integrative. Full affective range reported and expressible; injustice nameable alongside acceptance; practice has accompanied attempts to change circumstances; Ferrer criteria evidenced over time.
Archetypal identification vs. individuation	Total script. Identification described as constitutive rather than chosen; no source or alternative nameable; self-advocacy framed as lapse; direct questions about desire return blankness.	Partial. Identification recognized as taught but largely unquestioned; self-advocacy possible with accompanying guilt; preferences reportable with prompting.	Reflective agency. Identification held as one valued aspect among several; its cultural origin nameable; self-advocacy exercised without framing it as failure; desires reportable directly.

The anchors are descriptive reference points intended to guide clinical inquiry and the development of items. They are not a validated scoring system.

The hardest of these to apply is the desire-access indicator on Dimension 3, which asks the assessor to distinguish desire that has been renounced from desire that never formed. Dhawan’s (2005) clinical observations suggest the distinction is discernible, but it collapses easily, and it collapses in both directions: reading renunciation as absence pathologizes a woman who has genuinely let something go, while reading absence as renunciation credits a development that never occurred. The questions themselves are openings for exploration rather than items. They have not been piloted, and using them in research would require substantial revision, including translation and back-translation into the relevant regional languages.

### 5.2 Dimension 1: agency gradient

The Agency Gradient is based on the premise that spiritual engagement exists on a continuum, from practices that are freely chosen and reflectively embraced to those shaped by normative pressure and social constraints. As per the context examined here, most forms of spiritual engagement are likely to fall somewhere between these two endpoints. A practice may be taken up in response to social expectations while still holding genuine personal meaning, or it may be acquired through socialization and gradually become integrated into one’s sense of self.

Consequently, the assessment focuses not on the content of a woman’s beliefs but on the conditions under which her beliefs and practices were adopted. The key question is the extent to which she was able to decline, defer or modify her spiritual engagement without experiencing significant social, interpersonal, or psychological costs. However, one important caution is that constrained agency, by itself, does not provide evidence of spiritual bypassing. A practice entered into under obligation may still foster genuine transcendence, and coercion at the point of entry says little, on its own, about what that practice later comes to mean. The Agency Gradient therefore situates a woman along a single dimension; it does not, by itself, establish a diagnosis.

### 5.3 Dimension 2: functional directionality

Functional Directionality asks whether spiritual engagement moves a person toward psychological integration, emotional access, and interpersonal authenticity, or away from unresolved grief, legitimate grievance, and developmental growth. This dimension draws on Ferrer’s (2002) participatory criteria, according to which authentic spiritual engagement tends to foster greater moral awareness, self-knowledge, and compassion. It also engages with, though is not reducible to, Pargament’s (1997) functional distinction between positive and negative religious coping—a distinction concerned not with what a person believes, but with the psychological function those beliefs serve. A culturally adapted assessment tool would therefore need to include items addressing karma-based acceptance of structural injustice, the spiritual delegitimization of anger, and the use of *seva* to justify self-neglect, none of which are represented in the current SBS-13.

### 5.4 Dimension 3: archetypal identification versus individuation

The third dimension assesses the degree to which a woman has internalized mythological-religious identity scripts as totalizing self-definitions, as opposed to achieving reflective critical distance and an individuated sense of self. Drawing on Rowland (2002) and Young-Eisendrath (1997), both of whom argue that individuation cannot be understood independently of the gendered archetypal systems within which a woman is formed, the clinically relevant question is not whether a woman identifies with figures such as Sita or the pativrata ideal, but whether that identification has been consciously examined and appropriated as one partial dimension of a richer selfhood, or whether it constitutes a totalizing definition that forecloses desire, protest, and self-advocacy. As indicated above, Daniels’ (2005) multi-vector approach offers a theoretical model from which these two approaches can be distinguished from one another without assuming any normative development. A woman who has reflectively appropriated aspects of Sita’s steadfastness while retaining the capacity for anger, critical reflection, and self-advocacy occupies a fundamentally different clinical position from one for whom such identification is total, unexamined, and sustained through social enforcement. The former individual might be expressing an agentic and spiritually significant identification of herself within her differentiated self-structure. The latter, on the other hand, might be functioning under an oppressive model of identification whereby she limits her own potential emotional responses.

### 5.5 Conceptual distinctiveness from adjacent constructs

A new framework earns its place only if it accounts for something that existing constructs do not. Several established constructs overlap substantially with the phenomena described here, and the case for the present framework rests on identifying what each leaves unaddressed. The comparisons that follow are conceptual rather than empirical. Establishing incremental validity would require demonstrating that the three proposed dimensions account for variance in psychological outcomes beyond that explained by existing measures. That task falls outside the scope of the present paper and is deferred to the research programme outlined later.

Among existing constructs, self-silencing (Jack, 1991) is the closest conceptual analogue, and recent research suggests that it is particularly relevant to the population examined here. In a sample of 304 Indian women, Iqbal et al. (2025) found that family conformity orientation predicted self-silencing, which in turn predicted depression. The overlap with the present account is therefore substantial. However, self-silencing theory does not offer the legitimating apparatus that the present framework requires. In Jack’s formulation, a woman suppresses to preserve a relationship, and the cost of that suppression is available to her as a cost even when she chooses to bear it. The mechanisms described here operate differently: suppression is reframed as spiritual accomplishment, which means the woman may have no vocabulary in which to register it as a loss at all. Silencing that is understood as sacrifice and silencing that is understood as attainment are phenomenologically distinct, and only the second raises the assessment problem this framework addresses — that the clinician and the client may both read the same self-effacement as virtue.

Experiential avoidance and psychological inflexibility, as conceptualized within acceptance and commitment approaches, describe the unwillingness to remain in contact with aversive internal experience and the behavioral narrowing that follows. Functional Directionality clearly draws on this logic. The complication is that acceptance-based frameworks treat acceptance of unwanted experience as therapeutic, whereas the difficulty in the cases discussed here is that a culturally supplied acceptance is already in place and may itself be doing the avoiding. Distinguishing willing acceptance from prescribed acquiescence requires attention to how the acceptance was arrived at, which is what the Agency Gradient contributes and what a measure of experiential avoidance alone would not register.

Religious coping is the most developed alternative, and here the paper must acknowledge a resource it has so far underused. Tarakeshwar et al. (2003) developed a measure of religious coping specifically for Hindus, finding it a salient construct related to better mental health — which weakens any claim that Pargament’s framework simply lacks Hindu application. The distinction the present framework draws is nonetheless a different one. Positive and negative religious coping are differentiated largely by the valence of the person’s relationship to the sacred: negative coping involves spiritual discontent, punishing attributions, felt abandonment. What is described here is not discontent but its opposite. A woman whose beliefs about karma foreclose the expression of anger may report a wholly benevolent, collaborative relationship with the divine and would likely be classified as a positive religious coper. Whether a spiritual framework expands or constrains psychological possibility is orthogonal to whether it is experienced as supportive, and it is this orthogonal dimension that the present framework seeks to capture.

Expressive suppression, as conceptualized within Gross’s (1998) process model, captures the regulatory strategy but is deliberately agnostic about its content and justification. For instance. Two women may receive the same scores on measures of expressive suppression while differing considerably in whether their suppression is freely chosen, what makes it legitimate, and whether they experience it as restrictive or as a sense of accomplishment. Suppression scores alone may not be able to capture these differences, although having an important implication for clinical formulation. Similarly, existing measures of spiritual bypassing face similar limitation. The SBS-13 (Fox et al., 2017) assesses psychological avoidance and spiritualizing as general tendencies but includes no items that address the specific mechanisms proposed here. Its two-factor structure also presupposes a self that is actively avoiding psychological experience, whereas the Archetypal Identification dimension treats the status of that self as an empirical question rather than an *a priori* assumption.

Taken together, these established constructs each illuminate an important aspect of the phenomenon while taking for granted what the others leave unresolved. Measures of self-silencing assume that the psychological cost is accessible to conscious awareness. Acceptance-based models assume that acceptance, where present, reflects a genuine choice. Religious coping measures assume that the emotional valence of religious experience corresponds to its psychological function. Measures of expressive suppression, in turn, leave the question of how suppression is justified unanswered. The three dimensions proposed here are intended to keep these questions open simultaneously, rather than resolving them in advance.

### 5.6 Discriminating cases

The claim that this framework captures phenomena beyond existing constructs is best evaluated by considering cases in which its dimensions diverge from those assessed by established measures. The three illustrative profiles presented below are valuable because each points to a pattern of clinical judgment that conventional instruments would be unlikely to detect.

Consider, first, a woman who reports no subjective conflict about her domestic self-effacement, experiences her relationship with the divine as wholly supportive, and shows no elevation on measures of expressive suppression because there is little consciously experienced affect to suppress. She scores low on measures of self-silencing, as the construct assumes an internal division in which an authentic voice is consciously restrained, and she reports no such conflict. She likewise scores low on measures of negative religious coping, since she expresses neither spiritual distress nor punitive views of the divine, nor any sense of divine abandonment. On the SBS-13, she may also fall within the normative range because, from her own perspective, she is not using spiritual language to avoid psychological distress. Each existing measure therefore returns a largely benign interpretation. The present framework, however, arrives at a different conclusion. She occupies the constrained end of the Agency Gradient, the avoidant end of Functional Directionality, and the total-script end of Archetypal Identification. Taken together, this profile is clinically significant precisely because the absence of reported conflict is itself diagnostically meaningful. Existing constructs struggle to identify such a case because they assume that psychological cost is available to conscious awareness.

The second profile runs the other way. A woman with a long contemplative practice scores high on expressive suppression and high on self-silencing: she does withhold, deliberately and often. The established reading is concerning. The framework reads her differently- high on the Agency Gradient, since she has altered and suspended the practice at various points and can say what it does for her; integrative on Functional Directionality, since anger and grief remain available to her and the practice has accompanied attempts to change her circumstances; and reflective on the third dimension, since she holds her commitments as chosen rather than constitutive. Here the framework prevents a false positive that the suppression and self-silencing measures would generate.

The third concerns two women with identical SBS-13 scores. For one, spiritualizing operates as an individual defense against material she has encountered and turned from, which is the phenomenon Welwood described. For the other, the same score reflects a collective script absorbed before any such encounter took place. The scores converge; the mechanism does not, and neither does the indicated response. The first calls for work on what is being avoided; the second calls for work on whether a self-capable of avoiding was permitted to form.

These cases are constructed rather than observed, and they establish a conceptual claim rather than an empirical one: that there are clinically consequential distinctions the established constructs are not built to draw. Whether the dimensions also add predictive variance beyond those constructs is a separate question, and one the psychometric program described below would settle.

## 6 Applied implications

### 6.1 Clinical practice

For clinicians working with clients from these communities, the theoretical framework suggests a particular need for developing a new level of competence, which could be called spiritual bypassing literacy: the ability to discern psychologically functional forms of spirituality from dysfunctional spiritual processes. Cultural artifacts such as karma-based explanations of hardship, expressions of spiritual acceptance, or even seva as justification for subordinating oneself are all quite normative manifestations within the cultural milieu and are thus likely to be interpreted by clinicians as appropriate forms of religious coping. Brown’s (1994) feminist therapy theory provides a valuable procedural framework that, augmented by transpersonal sensibilities, would allow a clinician to integrate the importance of spirituality in these clients’ lives while still paying attention to its possible defensive functions. The goal is not to challenge clients’ commitment to religion, but rather to provide a safe forum wherein the psychological functions of their spiritual life can be examined and, where necessary, adjusted.

To illustrate the proposed model in action, let us examine a hypothetical case vignette. An Indian woman comes into therapy, and during the first few sessions, she consistently explains her situation of having a troubled marriage in terms of karma and how it dictates the way that she suffers quietly, thereby proving her spiritual strength. To a clinician unfamiliar with the above theory, such statements are easily mistaken for culturally appropriate attempts to make sense out of her situation; but they may well serve other functions too.

When the client’s place on the Agency Gradient is examined, it turns out that this religious interpretation of her life experiences has never been chosen or consciously decided upon; she has simply absorbed it through familial socialization, from a very young age onwards. In other words, there has been no stage of reflection, evaluation, modification, or rejection on her part. At once, one becomes uncertain about the appropriateness of distinguishing Archetypal Identification from Individuation. When asked about her female role models, this client is quite confused by the question, since making such a distinction implies choice, which has never entered into consideration from her point of view.

Indeed, in this case, the interaction between Dimensions 1 and 3 becomes clinically important: in a case of this kind, constrained agency and an unexamined, comprehensive archetypal identification appear to sustain one another, though the vignette illustrates the pattern rather than evidencing it. The goal here is not to encourage the rejection of any spiritual commitments, but to enhance a sense of reflexivity to help the client identify culturally imposed scripts, think about alternative interpretations of reality, and discriminate between authentic spiritual transcendence and psychologically harmful self-denial. The evaluation of Functional Directionality makes it clear that there is limited ability to express one’s feelings such as anger, grief, and self-advocacy, and negative emotions are always reframed as karma to prevent conscious recognition and processing. All taken together, this three-dimensional profile suggests structural spiritual bypassing and implies that clinical interventions should start with opening access to affective processes and promoting identity differentiation before addressing spiritual beliefs specifically.

### 6.2 Ethical safeguards for clinical use

A framework of this kind carries an obvious hazard. Used carelessly, it hands a clinician a vocabulary for reinterpreting a client’s religious life as pathology — and the vocabulary is persuasive enough that the reinterpretation may feel like insight to both parties. What follows are conditions for its use.

Spiritual acceptance is not evidence of avoidance. Equanimity before what cannot be changed is a recognized good in most contemplative traditions and in a good deal of clinical practice besides. The concern here is narrower: acceptance that forecloses an inquiry the client would otherwise have wanted to pursue. That cannot be read off the acceptance itself, which is why the Agency Gradient and Functional Directionality are assessed separately, and why both can return benign readings.

The client, rather than the clinician, remains the primary authority in interpreting her experience. The questions in Table 1 are designed to support a collaborative process of exploration, not to be privately scored by the clinician or used to make unilateral judgments about the client. A woman is ultimately better placed than her therapist to determine whether a particular spiritual practice expands or limits her psychological possibilities. When the client’s understanding differs from that of the clinician, this difference should first be considered with respect to the therapeutic relationship rather than treated as evidence of the client herself.

This principle becomes especially important when the clinician’s own commitments differ from those of the client. A secular therapist, a devout therapist, and a feminist therapist may each approach a woman’s self-sacrifice through a different interpretive lens, and none of these perspectives is free from values. The therapeutic task, therefore, is to expand the client’s range of psychological possibilities rather than to reshape her beliefs. A favorable outcome is one in which anger, grief, and self-advocacy become available as legitimate responses, and in which she is able to reflect critically on her spiritual commitments rather than inhabit them unreflectively. It is not one in which those commitments have necessarily been revised or abandoned. Treating continued religious observance as evidence of therapeutic failure substitutes the clinician’s personal commitments for clinical judgment.

The framework should likewise be applied only where its scope conditions are met. The Vaishnava-inflected material discussed in this paper may not readily generalize to clients from different religious traditions, regional contexts, or class positions. In such cases, the appropriate clinical response is inquiry rather than extrapolation. The work itself also carries costs that should be recognized rather than treated as obstacles to intervention. Helping a woman recognize a cultural script as a script may have familial, marital, and material consequences, and she is the one who will bear them. The pace of that work should therefore remain hers to determine, and those potential costs should form part of the therapeutic conversation rather than remain outside it.

### 6.3 Psychometric development and future research

The SBS-13 (Fox et al., 2017) does not capture the culturally specific channels through which spiritual bypassing appears to operate in the population described here. A culturally adapted measure would require iterative development: theoretical construct mapping, qualitative item generation with Indian women across demographic groups, expert review, pilot testing, and confirmatory factor analysis. The three-dimensional framework proposed in Table 1 provides a preliminary construct map for that process.

The model generates testable propositions, and validating it will likely require moving through qualitative, quantitative, and mixed-methods stages rather than any single design. As a starting point, semi-structured interviews with Hindu women across different regions, castes, and generational cohorts could establish whether the Agency Gradient, Functional Directionality, and Archetypal Identification dimensions are recognizable in women’s own accounts of their spiritual lives, and whether the language used here to describe them fits or needs revision. Phenomenological research could explore first-person experiences across the three dimensions in more depth. Longitudinal research could examine the relationships between the proposed mechanisms and psychological outcomes such as depression, somatization, and identity diffusion. A mixed-methods sequence — qualitative item generation followed by pilot testing and confirmatory factor analysis, as outlined earlier in this section — would be the natural route toward a validated instrument, and would also allow the three dimensions to be tested for measurement invariance across caste, class, religious tradition, and region, rather than assumed to hold uniformly. Comparative work building on Motiño et al. (2021) could examine whether the three dimensions show differential patterns across these same groups within India — a particularly important question given the framework’s current restriction to urban, middle-class Hindu women in Vaishnava-influenced contexts. The Indian Psychology movement (Cornelissen et al., 2014) is well-positioned to advance this agenda, but feminist methodology must become central to that effort rather than peripheral. Adaptation of Pargament’s religious coping framework to Hindu philosophical contexts — developing Indian-specific criteria for what constitutes positive and negative religious coping is a particularly tractable empirical project that could proceed in parallel.

## 7 Discussion

This paper has argued that spiritual bypassing, among the women whose contexts it examines, may be a structured, culture-specific, and gendered phenomenon that requires not merely a critique of existing Western transpersonal models but the development of a new one. The paper’s contributions fall into three areas.

First, it contributes to the decolonization of transpersonal psychology as a discipline (Patel, 2012). Welwood’s foundational construct embeds voluntarism, individualism, and an intrapsychic locus of analysis that reflect specific cultural assumptions rather than universal truths about spiritual experience. The revision proposed here keeps Ferrer’s (2002) participatory ground while showing that Wilber’s pre/trans distinction remains serviceable descriptively once stripped of its normative sequencing, and that Daniels’ (2005) multi-vector model supplies what a cross-cultural transpersonal psychology needs.

Second, this paper extends feminist psychology’s engagement with spirituality by viewing spirituality as a structural phenomenon rather than only a personal one. Brooks (2010) argued that feminist and transpersonal perspectives share a commitment to questioning conventional understandings of the self. They also suggested that bringing these perspectives together remains an underused resource for social transformation. The present argument builds on this insight by proposing that, in the contexts examined here, such an intersection is not merely valuable but necessary. Because spirituality is already shaped by social and political relations, it cannot be fully understood as a private or intrapsychic phenomenon. The three mechanisms proposed in this paper offer a culturally situated account of how gendered affect suppression may be spiritually legitimized and socially reproduced. In this way, the framework extends feminist psychological work on self-silencing (Jack, 1991) by proposing that spirituality may function not merely the context within which self-silencing unfolds, but as an interpretive framework through which self-silencing is enacted, moralized, and rendered largely invisible to those who experience it.

Third, in relation to Indian psychology, the present discussion helps to illustrate that spirituality cannot be conceptualized as a single cultural phenomenon with a consistent set of psychological effects. Instead, as Dube’s (1988) work indicates, the specific roles played by karma beliefs, seva values, and mythological identification in mediating gendered affect regulation are a product of complex sociocultural intersections—of caste, class, gender, region, and generation—that are not adequately captured by existing theory and research paradigms. Indeed, while recognizing Dube’s (1988) insight regarding the need to consider gender construction within Hindu patrilineal India as part of an ongoing history of socialization, the archetypal mechanism described here suggests that any interpretation of mythological scripts as abstract cultural tools that are separate from their socializing context is insufficient.

It is worth restating plainly what this framework is not arguing. None of the three mechanisms described above imply that karma, seva, or identification with figures such as Sita is inherently pathological. The Functional Directionality dimension exists precisely because spiritual engagement can move in different directions. The same belief in karma that forecloses a woman’s anger in one instance may, in another, provide the very means through which she is able to metabolize a loss for which she has no other language (Anand, 2009). The concern of the present paper is therefore not Hindu spirituality itself, but the more limited set of conditions under which spiritual meaning-making comes to suppress rather than process affect, and self-sacrifice is rendered unquestionable rather than freely chosen. This argument does not concern spiritual engagement that is adaptive, freely chosen, and psychologically integrative.

The counter-traditions discussed earlier further support this argument. Examining figures such as Mirabai, Akka Mahadevi, and Lal Ded does not undermine the critique presented here. Instead, these examples situate the critique within the wider interpretive landscape of Hindu traditions. Through their lives, spirituality and gendered identity have long been negotiated in different ways that cannot be understood as fixed in the form examined in this paper. An appropriate account of spiritual bypassing must therefore allow for both possibilities. Spiritual practice may reinforce self-erasure by sustaining existing expectations, but it may also provide a means of challenging those same expectations. The way spiritual practice functions in a particular case cannot be assumed in advance. Whether it contributes to self-erasure or becomes a means of resistance is ultimately an empirical question, rather than a general claim that can be made about the tradition.

### 7.1 Limitations

Several limitations should be acknowledged. As a conceptual contribution, this paper does not provide empirical evidence for the proposed mechanisms or dimensions. Future qualitative and quantitative research will be necessary to test and refine these propositions. This limitation applies not only to the theoretical model but also to the proposed assessment framework. The indicators presented in Table 1 and the anchors in Table 2 have not yet been piloted, and the empirical distinctiveness of the proposed poles has not been established. It also remains to be determined whether the three dimensions explain variance beyond established constructs such as self-silencing, experiential avoidance, and religious coping. Thus, the extent to which these dimensions represent distinct aspects of the proposed framework remains an empirical question. The framework is also limited by the nature of the literature on which it is based. As Vindhya (2007) notes, mainstream psychological research on gender in India has relied disproportionately on urban, middle-class samples. Consequently, a framework developed primarily from this literature may also reflect the limitations of this evidence base. The scope specified here therefore reflects not only a deliberate restriction but also the limits of the evidence currently available. The framework is explicitly restricted to Hindu women in urban, middle-class Indian contexts shaped by the Vaishnava pativrata ideal — its applicability to Muslim, Christian, Sikh, or Buddhist women, to Dalit and lower-caste communities, to rural populations, and to the Indian diaspora, is an open question that separate studies will need to address. Intersectionality, particularly along the axes of caste and class, has been acknowledged but not theoretically developed; this is a priority for subsequent work. The cross-cultural emotion regulation literature particularly findings from East Asian samples suggesting that expressive suppression may not carry the same psychological costs in all collectivist contexts (Matsumoto et al., 2008) introduces uncertainty into the paper’s claims about the harmful effects of spiritualized affect suppression. As argued above, the East Asian findings raise a question that requires India-specific empirical investigation rather than direct transfer; those data are not evidence about Hindu Indian women, and the specifically theological dimension of the spiritualized affect regulation mandate may modulate the effects of suppression in ways that general collectivism–individualism distinctions do not capture. Bem’s (1981) gender schema theory and Bourdieu’s (1977) concept of habitus were invoked to provide definitional boundaries for the construct of collective spiritual bypass scripts; fuller engagement with those frameworks, and with the broader cultural psychology of gender socialization, is a task for subsequent theoretical work.

## 8 Conclusion

Spiritual bypassing, as it appears among urban, middle-class Hindu women in Vaishnava-influenced contexts, is unlikely to be adequately understood through existing Western frameworks without substantial critical revision. Those frameworks presume a choosing subject, a boundaried self, and an intrapsychic mechanism of defense that sit uneasily with the structural realities documented in these women’s spiritual lives — realities in which spirituality is frequently imposed, self is relationally constituted, and bypassing is accomplished through collective, mythological, and systemic means. Three structural mechanisms through which this appears to occur have been proposed, and a three-dimensional framework has been operationalized through indicators and assessment questions, providing a foundation for an empirical research program.

The implications extend beyond Indian psychology. Transpersonal psychology cannot claim cross-cultural validity until it has reckoned with the structural conditions in which spirituality is constructed, constrained, and transformed. Feminist psychology is unlikely to achieve an adequate understanding of these women’s psychological lives without attending to the spiritually organized systems through which their emotions are regulated and their selfhood is shaped. And Indian psychology cannot claim theoretical maturity until it treats gender as a constitutive rather than peripheral variable in its accounts of spiritual and psychological life. The three-dimensional framework proposed in this paper comprising the Agency Gradient, Functional Directionality, and the Archetypal Identification–Individuation continuum, together with the constructs of collective bypass scripts and the spiritualized affect regulation mandate—provides an initial conceptual architecture for this undertaking. It is theoretically grounded and clinically applicable, while also being intended for empirical testing, refinement, and further elaboration through the research agenda advocated in the present paper.
